# Epidémiologie et facteurs de risque des complications respiratoires majeures après chirurgie de l'aorte abdominale au CHU Ibn Sina, Maroc

**DOI:** 10.11604/pamj.2013.14.13.1853

**Published:** 2013-01-08

**Authors:** Almahdi Awab, Brahim Elahmadi, Tarik Lamkinsi, Rachid El Moussaoui, Ahmed El Hijri, Abderrahim Azzouzi, Mustapha Alilou

**Affiliations:** 1 Université Mohammed V, unité de pédagogie et de recherche en anesthésie réanimation, CHU Ibn Sina, Rabat, Maroc; 2 Laboratoire de génétique et biométrie, Université Ibn Tofail, Kénitra, Maroc

**Keywords:** Complications respiratoires postopératoires, chirurgie de l'aorte, facteurs de risque, Postoperative respiratory complications, aortic surgery, risk factors

## Abstract

**Introduction:**

L'incidence des complications respiratoires postopératoires (CRPO) reste très diversement appréciées selon les critères diagnostiques retenues dans les différentes études, ce qui la fait varier de 5 à plus de 50%. Les CRPO majeurs après chirurgie de l'aorte abdominale sont responsables d'une grande morbi-mortalité pouvant aller jusqu’à 36%, d'une durée d'hospitalisation et d'un coût plus importants. Ainsi dans l'optique d'améliorer notre prise en charge périopératoire de la chirurgie de l'aorte, nous avons décidé de mener une étude pour dresser le profil épidémiologique et déterminer les facteurs de risque des complications respiratoires dans notre contexte

**Méthodes:**

Il s'agit d'une étude de cohorte rétrospective du mois de Janvier 2007 au mois de décembre 2011 portant sur l'ensemble des patients opérés pour pathologie aortique au bloc opératoire central de l'hôpital Ibn Sina de Rabat, Maroc.

**Résultats:**

Cent vingt cinq patients ont été inclus dans notre étude, 24 patients ont été opérés pour anévrysme de l'aorte abdominale et 101 patients pour lésion occlusive aortoiliaque. Dans notre série 22 malades soit 17,6% ont présenté une complication respiratoire majeure avec, une reventilation dans 4,8% des cas, une difficulté de sevrage de la ventilation artificielle dans 3,2% des cas, une pneumopathie dans 4% des cas, un syndrome de détresse respiratoire aigue (SDRA) dans 4% des cas et une nécessité de fibroaspiration bronchique dans 1,6% des cas. En analyse univariée: l’âge, la présence d'une BPCO avec dyspnée stade 3 ou 4, la présence d'une anomalie à l'EFR préopératoire, la présence d'un stade avancé (III ou IV) de LOAI et la reprise chirurgicale étaient statistiquement associés à la survenue d'une complication respiratoire postopératoire. En analyse multivariée, seule une anomalie à l'EFR en préopératoire constituait un facteur de risque indépendant de survenue d'une complication respiratoire postopératoire dans notre série avec un Odds Ratio (OR): 11,5; un Intervalle de Confiance (IC) à 95% de (1,6 - 85,2) et un p = 0,016.

**Conclusion:**

Au terme de notre étude, il nous parait donc nécessaire pour diminuer l'incidence des CRPO majeurs dans notre population, d'agir sur les facteurs que nous jugeons modifiables tel l'amélioration de l’état respiratoire basal moyennant une préparation respiratoire préopératoire, s'intégrant dans un véritable programme de réhabilitation et associant une rééducation à l'effort, une kinésithérapie incitative ainsi qu'une optimisation des thérapeutiques habituelles.

## Introduction

La chirurgie de l'aorte abdominale est responsable d'une mortalité de 5 à 8% en dehors de l'urgence et elle est grevée de complications graves dans 20% des cas [1, 2]. Les complications respiratoires après chirurgie de l'aorte ont très largement été étudiées ces dernières décennies. Leur incidence reste très diversement appréciées selon les critères diagnostiques, ce qui la fait varier de 5 à plus de 50% [3]. Plusieurs facteurs pré, per et postopératoires ont été identifiés dans la littérature comme favorisant la survenue des complications respiratoires (CRPO) majeurs [1, 3].

Ces dernières sont responsables, d'une morbi-mortalité pouvant aller jusqu’à 36%, d'une durée d'hospitalisation et d'un coût plus importants [1, 4]. Ainsi dans l'optique d'améliorer notre prise en charge périopératoire de la chirurgie de l'aorte, nous avons décidé de mener une étude pour dresser le profil épidémiologique et déterminer les facteurs de risque des complications respiratoires dans notre contexte.

## Méthodes

Il s'agit d'une étude de cohorte rétrospective réalisée, après avis du comité d’éthique local de la faculté de médecine et de pharmacie de Rabat (FMPR), du mois de Janvier 2007 au mois de décembre 2011 portant sur l'ensemble des patients opérés pour pathologie aortique au bloc opératoire central de l'hôpital Ibn Sina de Rabat. Plusieurs paramètres étaient recueillis: l’âge, le sexe, les antécédents médicaux et chirurgicaux, les résultats du bilan préopératoire, le déroulement de l'acte opératoire et l’évolution postopératoire.

L'exploration préopératoire comportait systématiquement: un électrocardiogramme, une radiographie thoracique, une exploration fonctionnelle respiratoire (EFR), une échocardiographie, et une échographie des troncs supra-aortiques. Tous les malades ont bénéficié d'une anesthésie générale associant un hypnotique, un morphinique et un curare pour l'induction, puis isoflurane et morphinique pour l'entretien, l'incision était xypho-pubienne.

Le monitorage hémodynamique peropératoire était basé sur la mesure invasive de la pression artérielle et la pression veineuse centrale. La ventilation artificielle per et postopératoire était standardisée pour tous les patients, avec un volume courant de 6 à 8ml/kg sans dépasser une pression de plateau de 30 cm H2O, une fréquence respiratoire à 12 cycles/min et une pression expiratoire positive (PEP) ne dépassant pas 55cm H2O. Tous les malades étaient systématiquement admis en unité de soins intensifs (USI).

Les définitions suivantes ont été retenues pour notre étude: La bronchopneumopathie chronique obstructive (BPCO) et l'insuffisance respiratoire chronique étaient définies selon les recommandations pour la pratique clinique mises à jour en 2009 [5]. La sévérité clinique de la BPCO était définie en fonction de l’échelle de Sadoul modifiée [5]. L'EFR était considérée pathologique pour toute valeur inférieure à la normale du VEMS, CV, Tiffeneau, DEM 25, DEM 50 et DEM 75. Les complications respiratoires postopératoires majeures ont été définies par l'existence de l'une ou plus des situations suivantes: une difficulté de sevrage de la ventilation artificielle après plus de 48 heures, la nécessité d'une reventilation invasive ou non invasive, et l'apparition d'un syndrome de détresse respiratoire aiguë (SDRA), d'une pneumopathie infectieuse ou d'une macro-atélectasie nécessitant une fibroaspiration. L'infarctus du myocarde (IDM) répondait à la définition universelle de 2007 [6]. Le dosage de la Troponine I a été systématiquement réalisé à la 12^ème^; heure de l'intervention, ou dans le postopératoire immédiat en cas d'instabilité hémodynamique et de troubles de la repolarisation sur l’électro-cardiogarmme, puis chaque jour jusqu’à la normalisation. L'insuffisance rénale répondait à la classification de RIFLE [7].

Notre critère de jugement principal était la survenue d'une complication respiratoire majeure, les critères de jugement secondaires étaient la survenue d'une autre complication. L'analyse statistique a été réalisée à l'aide du logiciel SPSS 13. Les variables quantitatives ont été exprimées en moyennes± déviations standard ou médiane et quartiles, et les variables qualitatives en effectifs et pourcentages. Les variables quantitatives ont été comparées par le test t de Student pour échantillons indépendants ou le test U de Mann-Whitney quand la distribution est non gaussienne, et les variables qualitatives ont été comparées à l'aide du test de Chi 2 de Pearson ou du test exact de Fisher. Une erreur de première espèce était considérée comme significative pour un p

L'analyse multivariée a fait appel à la régression logistique sur un modèle incluant les paramètres dont le p était inférieur ou égal à 0,2 en analyse univariée.

Ce travail a été approuvé par la commission d’éthique de la faculté de médecine et de pharmacie de Rabat, Maroc.

## Résultats

Cent vingt cinq patients ont été inclus dans notre étude, 24 patients ont été opérés pour anévrysme de l'aorte abdominale (AAA) et 101 patients pour lésion occlusive aortoiliaque (LOAI). Les caractéristiques épidémiologiques, cliniques et paracliniques sont représentées dans le Tableau 1.

**Tableau 1:** Caractéristiques de la population étudiée

Caractéristiques	Valeurs
Age (ans)	58,6 ± 11,5
**Sexe**	
Hommes	119 (95%)
Femmes	6 (5%)
**ASA**	
I	56 (44,8%)
II et III	69 (55,2%)
**Antécédents**	
HTA	29 (23,2%)
tabagisme	109 (87,2%)
Diabète	16 (12,8%)
Cardiopathie ischémique	12 (9,6%)
BPCO (dyspnée stade 1 ou 2)	27 (21,6%)
BPCO (dyspnée stade 3 ou 4)	5 (4%)
**Pathologie aortique**	
LOAI	101 (81%)
AAA	24 (19%)
**Stade de la LOAI**	
I et II	17 (17%)
III et IV	84 (83%)
Anomalies à l'EFR	30 (24%)
Durée d'intervention (min)	258 ± 70
Durée de clampage (min)	35 ± 15
Saignement peropératoire (ml)	500 (200-900)
Apports liquidiens peropératoires (ml)	5595 ± 1563
Séjour en unité de soins intensifs (jours)	2 (1-3)
Mortalité (toute cause)	21 (16,8%)

Les variables quantitatives sont exprimées en moyenne ± écart type quand la distribution est gaussienne ou en médiane (interquartile) si elle est non gaussienne. Les variables qualitatives sont exprimées en effectif (pourcentage). AAA : anévrysme de l'aorte abdominale ; ASA : classification de l'american society of anaesthesiologists ; BPCO : broncho-pneumopathie chronique obstructive ; F : femmes ; H : hommes ; HTA : hypertension artérielle ; LOAI : lésion occlusive aorto-iliaque

Dans notre série 22 malades soit 17,6% ont présenté une complication respiratoire majeure avec, une reventilation dans 4,8% des cas, une difficulté de sevrage de la ventilation artificielle dans 3,2% des cas, une pneumopathie dans 4% des cas, un syndrome de détresse respiratoire aigue (SDRA) dans 4% des cas et une nécessité de fibroaspiration bronchique dans 1,6% des cas. L'ensemble des complications postopératoires est représenté sur le Tableau 2. O[ugrave] on note, un taux élevé de reprises chirurgicales (14,4%), d'insuffisance rénale aigue (13,5%) et de syndrome d'ischémie-reperfusion (8,8%).

**Tableau 2:** Les complications postopératoires

Complications	valeurs
**Complications respiratoires majeures**	**22 (17,5%)**
Difficultés de sevrage	4 (3,2%)
Re-ventilation	6 (4,8%)
Pneumopathie	5 (4%)
SDRA	5 (4%)
**Macro-atélectasie nécessitant une fibroaspiration**	**2 (1,6%)**
Atélectasies	13 (10,4%)
**Autres complications**	****
Reprise chirurgicale	18 (14,4%)
Insuffisance rénale	17 (13,5%)
Ischémie-reperfusion	11 (8,8%)
Infarctus du myocarde	7 (5,6%)
Ischémie colique	3 (2,4%)
AVC ischémique	2 (1,6%)
Cholécystite alithiasique	2 (1,6%)

AVC : accident vasculaire cérébral ; SDRA : syndrome de détresse respiratoire aiguë

La comparaison des principaux éléments cliniques, paracliniques, et évolutifs entre les patients qui ont présenté (groupe I) et ceux qui n'ont pas présenté (groupe II) une complication respiratoire majeure, figure sur le Tableau 3. En analyse univariée: l’âge, la présence d'une BPCO avec dyspnée stade 3 ou 4, la présence d'une anomalie à l'EFR préopératoire, la présence d'un stade avancé (III ou IV) de LOAI et la reprise chirurgicale étaient statistiquement associés à la survenue d'une complication respiratoire postopératoire.

**Tableau 3:** Tableau comparatif des principaux éléments cliniques, paracliniques, et évolutifs entre les patients qui ont présenté une complication respiratoire majeure (Groupe I) et ceux qui n'ont pas présenté de complication respiratoire majeure (Groupe II)

Caractéristiques	Groupe I 22 (17,5%)	Groupe II 103 (82,5%)	p
Age (ans)	64 ± 9	57,8 ± 11,7	0,04
**Sexe**	****	****	****
Homme	20 (91%)	98 (95%)	NS
Femme	2 (9%)	5 (5%)	
Antécédents cardiovasculaires	7(31,8%)	31(30,1%)	NS
Tabagisme	20(90%)	89(86,4%)	NS
BPCO (dyspnée stade 3 ou 4)	3(13,6%)	2(1,9%)	0,01
ASA II et III	10 (44%)	49 (48%)	NS
Anomalie à l'EFR	15(68,2%)	15(13,6%)	0,016
LOAI avancé (Stade III ou IV)	22 (100%)	81(78,6%)	<0,001
Durée de l'intervention (min)	270 ± 80	250 ± 70	NS
Saignement peropératoire (ml)	900 [300-1200]	500 [200-800]	0,08*
Apports liquidiens per-op (ml)	5818 ± 1952	5544 ± 1481	NS
Séjour en réanimation (jours)	6,1 ± 3,3	1,9 ± 1,4	<0,001
Reprise chirurgicale	7(31,8%)	11(10,7%)	<0,001
Mortalité	6(27,3%)	15(14,6%)	NS

Les variables quantitatives sont exprimées en moyenne ± écart type et les variables qualitatives en effectif et pourcentage n (%).

* test U de Mann and Whitney

EFR : exploration fonctionnelle respiratoire ; groupe C : groupe avec complications respiratoires ; groupe T : groupe sans complications respiratoires; LOAI : lésion occlusive aorto-iliaque; NS : non significatif.

La durée d'intervention, le saignement et le remplissage vasculaire peropératoires, étaient plus importants dans le groupe I, sans que la différence entre les deux groupes ne soit statistiquement significatif. D'autres part, la durée de séjour en réanimation était significativement plus élevée dans le groupe I et le taux mortalité était aussi plus élevé dans le groupe I (27,3% Vs 14,6%) sans être statistiquement significative.

En analyse multivariée, seule une anomalie à l'EFR en préopératoire constituait un facteur de risque indépendant de survenue d'une complication respiratoire postopératoire dans notre série avec un Odds Ratio (OR): 11,5; un Intervalle de Confiance (IC) 95% de [1,6 – 85,2] et un p = 0,016.

## Discussion

L'incidence des CRPO reste très diversement appréciées selon les critères diagnostiques retenues dans les différentes études, ce qui la fait varier de 5 à plus de 50% [3]. Quand on ne prend en considération que les complications majeures, comme elles sont définies dans notre étude, leur incidence devient moindre allant de 5 à 25% [1, 3]. L'incidence des CRPO majeurs dans notre étude était de 17,6% ce qui reste élevé par rapport aux centres performants. Ceci est principalement tributaire de certaines particularités de notre population, à savoir la grande proportion de malades porteurs d'une BPCO (25,6%, p = 0,01) et dont 18,5% sont au stade 3 ou 4 de Sadoul, et puis des stades très évolués (III et IV) de la maladie occlusive aorto-iliaque (83%, p<0,001).

La présence d'une BPCO augmente significativement le risque de complications respiratoires postopératoires (atélectasie, broncho-pneumopathie, ventilation prolongée) en le multipliant par un facteur pouvant aller jusqu’à 5 [1, 8, 9]. Par ailleurs, dans notre série 87,2% de nos patients étaient fumeurs, et des études ont démontré que le tabagisme est un facteur de risque indépendant de survenue des CRPO même en l'absence de pathologie respiratoire associée [8]. Ceci justifierait dans notre contexte la nécessité d'un sevrage tabagique d'au moins huit semaines et d'une préparation respiratoire préopératoire, s'intégrant dans un véritable programme de réhabilitation et associant une rééducation à l'effort, une kinésithérapie incitative ainsi qu'une optimisation des thérapeutiques habituelles, comme proposé par Sutherland et al. [10].

Dans notre étude, nous avons trouvé qu'une anomalie à l'EFR était indépendamment associée à la survenue de CRPO avec un OR: 11,5; IC 95%: 1,6-85,2; p = 0,016. L'utilité des épreuves fonctionnelles respiratoires dans l’évaluation préopératoire reste un sujet très controversé. De rares études ont montré une corrélation entre l'existence d'une anomalie à l'EFR et la survenue de complications respiratoires postopératoires, mais toutes étaient sur un faible effectif de malades. D'autres études ont démontré que les données de l'EFR ne sont ni superposables ni supérieures à l'histoire clinique du patient [11]. Ceci à notre sens pourrait être justifié pour différents types de chirurgie avec lesquelles l’état clinique respiratoire reste évaluable en préopératoire, alors que pour des patients artéritiques à des stades évolués et dont le périmètre de marche est réduit, l'intérêt de la clinique seule dans l’évaluation respiratoire reste très discutable. Et comme affirme certaines études, un terrain o[ugrave] plusieurs facteurs d'atteinte respiratoire (tabagisme, BPCO, âge) sont souvent associés, l'intérêt de l'EFR peut être primordial pour détecter une anomalie infra clinique et optimiser la prise en charge préopératoire [11, 12].

L’âge avancé est fréquemment discuté dans la littérature comme facteur de risque de survenu de complications respiratoires postopératoires. On considère souvent qu'il n'est pas un facteur de risque indépendant si l'on tient compte des comorbidités qui y sont associées. Cependant, certaines études bien menées suggèrent un rôle propre de l’âge, dès 50 ans et risque augmente surtout après 70 ans [1, 2, 13–15]. Dans notre étude, la moyenne d’âge des patients ayant présenté une CRPO était de 64 ± 9 ans, qui était significativement différente avec un p = 0,04. Le stade avancé de la maladie artérielle de nos patients (stade III et IV), était significativement associé à la survenue des CRPO majeurs avec un p13,^16^, ^17^].

La voie d'abord, le plus souvent xypho-pubienne, joue un rôle considérable dans la genèse des complications respiratoires. L'intérêt préventif d'une incision transversale ou retro-péritonéale est cependant controversé [18]. Les techniques d'angioplastie endoluminales pour les AAA seraient responsables de moins de complications respiratoires [19–22]. L'analgésie est un des facteurs sur lequel il est possible d'agir dans le but de prévenir les complications respiratoires. Tous nos patients ont bénéficié du même protocole d'analgésie dans le postopératoire et le caractère rétrospectif de notre étude ne nous a pas permit de réaliser une analyse plus fine [23, 24].

Le taux de mortalité élevé rapporté dans notre série (16,8%) comparé aux données de la littérature [1, 4] est très probablement en partie lié aux particularités de notre population (BPCO, comorbidités et stades évolués de la maladie artérielle), mais aussi au manque d'expertise du moment o[ugrave] on réalise en moyenne 31 intervention/an (125 interventions/4ans

## Conclusion

Au terme de notre étude: l'incidence des CRPO était de 17,6%. L’âge, la présence d'une BPCO avec dyspnée stade 3 ou 4, la présence d'une anomalie à l'EFR préopératoire (facteur de risque indépendant), la présence d'un stade avancé (III ou IV) de LOAI et la reprise chirurgicale étaient statistiquement associés à la survenue d'une complication respiratoire postopératoire. Il nous parait donc nécessaire pour diminuer l'incidence des CRPO majeurs dans notre population, d'agir sur les facteurs que nous jugeons modifiables tel l'amélioration de l’état respiratoire basal moyennant une préparation respiratoire préopératoire, s'intégrant dans un véritable programme de réhabilitation et associant une rééducation à l'effort, une kinésithérapie incitative ainsi qu'une optimisation des thérapeutiques habituelles.
